# A multidisciplinary approach to screen the post-COVID-19 conditions

**DOI:** 10.1186/s12879-023-08006-4

**Published:** 2023-01-24

**Authors:** Nicola Squillace, Viola Cogliandro, Emanuela Rossi, Giuseppe Bellelli, Matteo Pozzi, Fabrizio Luppi, Maddalena Lettino, Maria Grazia Strepparava, Carlo Ferrarese, Ester Pollastri, Elena Ricci, Paolo Bonfanti, for the STORM Long-COVID Team, Giacomo Bellani, Andrea Biondi, Marina Elena Cazzaniga, Giuseppe Citerio, Ernesto Contro, Giuseppe Foti, Maria Grazia Valsecchi, Alban Rugova, Eleonora Maria Beretta, Marta Iannace, Anna Spolti, Valentina Orsini, Cristina Malafronte, Laura Valagussa, Daniela Ferlicca, Francesca Bettini, Valeria Bellin, Valeria Meroni, Mariangela Calabria, Stefano Gatti, Alfio Bronco, Claudio Ripa, Simone Sosio, Paola Faverio, Anna Monzani, Maria Cristina Ferrara, Cristina Zarcone, Carlo Ferrarese, Simone Beretta, Valerio Salvarani, Ornella Mauri, Carolina Da Re, Andrea Carrer

**Affiliations:** 1 Infectious Disease Unit, Fondazione IRCCS San Gerardo dei Tintori, Via GB Pergolesi, 33, Monza, Italy; 2 Bicocca Center of Bioinformatics, Biostatistics and Bioimaging, University of Milano-Bicocca, Monza, Italy; 3 Acute Geriatric Unit, Fondazione IRCCS San Gerardo dei Tintori, Monza, Italy; 4 Anesthesia and Intensive Care Unit, Fondazione IRCCS San Gerardo dei Tintori, Monza, Italy; 5 Respiratory Disease Unit, Fondazione IRCCS San Gerardo dei Tintori, Monza, Italy; 6 Cardiology Division, Fondazione IRCCS San Gerardo dei Tintori, Monza, Italy; 7 Clinical Psychology Unit, Department of Mental Health, Fondazione IRCCS San Gerardo dei Tintori, Monza, Italy; 8 Neurology Unit, Fondazione IRCCS San Gerardo dei Tintori, Monza, Italy; 9 School of Medicine and Surgery, University of Milano-Bicocca, Monza, Italy; 10 Fondazione ASIA, Buccinasco, Italy

**Keywords:** Post-COVID-19 conditions, COVID-19, Long-COVID, PASC, ICU

## Abstract

**Background:**

Post-COronaVIrus Disease 2019 (COVID-19) conditions (PCC) include multiple symptoms afflicting different organs and systems. To evaluate the frequency and type of them, we described our multidisciplinary approach with preliminary results of the first enrolled patients.

**Methods:**

We included patients aged ≥ 18 years with hospital admission for confirmed SARS-CoV-2 infection. Symptoms were grouped in five macro groups hereafter referred to as "Symptoms Category" (SC): respiratory SC (dyspnoea or cough), neurological SC (peripheral neuropathies, headache, impaired mobility, behavioural disorders), psychological SC (sleep disorders, mood disorders), muscular SC (arthromyalgia, asthenia), other SC (fever, alopecia, diarrhoea, weight loss, smell and taste alterations, sexual dysfunctions). SC were evaluated at discharge and at follow-up. Association between patients’ characteristics and presence of SC at follow up was estimated by a logistic multivariable regression model.

**Results:**

From June 2020 to July 2021, we followed up 361 patients: 128 (35.5%) who were previously admitted to Intensive Care Unit (ICU) and 233 patients to ordinary department. The median length of hospital stay was 20 days (Inter-Quartile-Range 13–32). Most patients (317/361, 87.8%) were still symptomatic at discharge, with one third referring three or more SC. At follow up, 67.3% (243/361) of patients still complained at least one SC. Moreover, 159 patients (44%) developed at least one new involved SC during follow up: 116 (72.9%) one SC, 39 (24.5%) two SC, 4 (2.5%) three or more SC. At follow up visit 130 of 361 (36%) were still with SC developed during follow up. At multivariable analysis presence of any SC at follow-up was associated with male gender (Odds Ratio [OR] 3.23, Confidence Interval [CI] 95% 1.46–7.15), ICU admission (OR 2.78, CI 95% 1.29–5.96) and presence of SC at discharge (OR 14.39, CI 95% 6.41–32.32).

**Conclusions:**

In our sample of patients with severe COVID-19, we found that PCC are highly variable and fluctuating over time; in particular, in about 50% of our patients new SC appear during follow up. Moreover, presence of PCC also in patients without SC at discharge and the variability of symptoms underlining the advisability of our multidisciplinary approach.

*Trial registration number:* ClinicalTrials.gov Identifier: NCT04424992, registered on 28 February 2020 https://www.clinicaltrials.gov/ct2/results?recrs=ab&cond=&term=NCT04424992&cntry=&state=&city=&dist  The current version of protocol is version 1.0 enrolling since June 2020. The enrollment is still ongoing.

## Background

Long-term sequelae, such as pulmonary dysfunction, psychological disorders, and reduced exercise capacity, were already described after 6 months of Severe Acute Respiratory Syndrome (SARS)-CoronaVirus (CoV) and Middle Eastern Respiratoy Syndrome-CoV infections [1]. Only months after the first COronaVIrus Disease 2019 (COVID-19) outbreak, multiple studies were published about the long-term consequences of SARS-CoV-2 infection, including persisting lung function impairment and central nervous system symptoms [2–8].

This condition of symptoms persistence, which can affect subjects of any age and with varying acute disease severity, has been recognized as a specific clinical entity called long-COVID, post-COVID-19 syndrome (PCS), or post-acute sequelae of SARS-CoV-2 infection (PASC), although there is not yet consensus on the appropriate definition [9, 10]. The National Institute for Health and Care Excellence (NICE) guidelines have proposed a clinical definition of the sequelae of COVID-19, in relation to the different temporal evaluations [11]. Recently World Health Organization (WHO) has tried, through a Delphi Consensus, to provide a clinical case definition and proposed to adopt the name of post-COVID-19 Conditions (PCC) [12].

Besides pulmonary damage, SARS-CoV-2 infection affects the cardiovascular system, the gastrointestinal tract, the central nervous system, and the kidney [13]. Direct viral toxicity, endothelial damage, and deregulated immune response could be responsible for these manifestations [13].

To date, different models for evaluating PCC have been adopted. As regards self-reported symptoms, most studies collected information by interviewing patients face-to-face [2–6], whereas some opted for telephone interviews [7, 8]. Other studies included physical examinations, such as chest X-Ray, pulmonary function, and blood testing, in all or some of the patients [2–5]. In addition, quality of life has been assessed using the Euro Quality- 5 Dimension-5 Level (EQ5D-5L) questionnaire on quality of life [14], neurocognitive sequelae using validated questionnaires [5, 15–18], and frailty using the Clinical Frailty Score (CFS) or Frailty index[17, 19].

Lombardy region was the epicentre of the first COVID-19 outbreak in Italy. As of 20 April 2022, the San Gerardo Hospital (Monza, Lombardy) admitted about 6133 patients with COVID-19. Of these, 952 (15.5%) died during hospitalization. Since a substantial part of COVID-19 survivors needed further medical attention, from June 2020 we set up a post-COVID clinic for patients who had been hospitalised in our centre.

The primary aim of this study is to describe a multidisciplinary approach for evaluating PCC, to assess the prevalence and the persistence of symptoms at least 3 months after discharge and to evaluate the risk factors associated with the persistence of these symptoms.

## Methods

### Post-COVID-19 conditions clinic model

In the usual model of care, patients with multiple health issues often book appointments for several exams and visits, most likely on different days. For the patients, this type of organization is time-consuming and expensive, and may frequently lead to neglect disorders that are perceived, rightly or wrongly, as less important, or that do not require immediate treatment.

In our model of care, survivors of hospitalization were contacted and invited to undergo an evaluation by our multidisciplinary team, composed of an infectious disease specialist, a pulmonologist, a geriatrician, an intensivist, a psychologist, a cardiologist, and a haematologist. The rationale of our approach is to optimize the management of both clinical and organizational aspects, investigating all potentially detrimental sequelae of COVID-19, using the design of a cohort study.

Since COVID-19 is a multisystemic disease, we hypothesized that a multidisciplinary approach was crucial to identifying all patient’s clinical problems and unmet needs. Initially, we included patients who had severe disease, defined as requiring mechanical ventilation (Intensive Care Unit [ICU] group). Afterward, we extended the enrolment also to those admitted to acute medical wards (Acute Medical Wards [AMW] group).

We excluded paediatric subjects (< 18 years old), pregnant patients and residents in long care facilities.

At least 3 months after hospitalization, all eligible patients were contacted by phone and invited to undergo a follow-up visit. Those who accepted were scheduled. A caregiver was allowed to accompany patients with cognitive or physical impairment.

Patients were enrolled from June 2020 to July 2021. Vaccination for SARS-Cov-2 was introduced in Italy in March 2021 and patients were usually vaccinated 6 months after infection. Because all the patients were observed at least after 3 months and within 5 months after the hospitalization no patient was vaccinated.

### Procedures

**Fig. 1:**
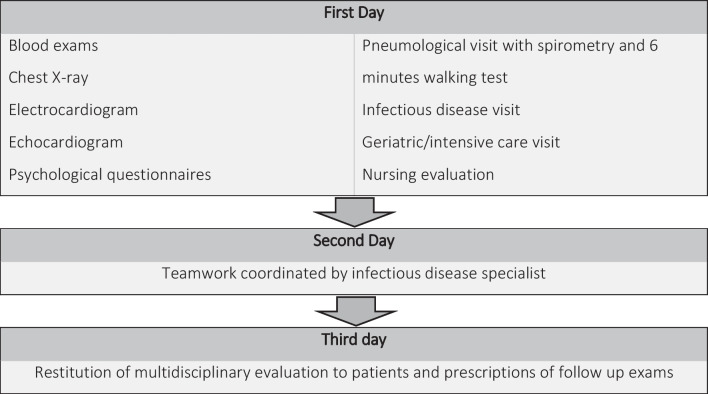
Organization of post COVID-19 clinic

The post-COVID-19 assessment is organized following an integrated care pathway (Fig. 1), as follows.

On the first day, all patients undergo the following assessments:

- blood exam (full blood count, liver function tests, coagulation tests with D-dimer, renal functions test, glycaemia, lactate dehydrogenase, C reactive protein, pro-brain natriuretic peptide), chest X-ray, electrocardiogram, transthoracic echocardiogram.
- measurement of Body Mass Index (BMI).
- self-administered psychological questionnaires, fulfilled by the patients or by caregivers including: Post-traumatic Symptom Scale (PTSS), Hospital Anxiety and Depression Scale (HADS), Generalized Anxiety Disorder Assessment (GAD), Clinical Outcomes in Routine Evaluation-Outcome Measures, Insomnia Severity Index, Depression Anxiety Stress Scale, Anxiety Scale Questionnaire, Five Facet Mindfulness Questionnaire, Coping Orientation to Problems Experienced-New Italian Version.
- infectious disease visit with physical examination. Past clinical history, symptoms present at the time of discharge and occurring during the months before the visit, demographical information, and ongoing symptoms are collected. The disease severity is defined as the highest grade of respiratory support received during hospitalization, on the following scale: level 1, not admitted to hospital with resumption of normal activities; level 2, not admitted to hospital, but unable to resume normal activities; level 3, admitted to hospital but not requiring supplemental oxygen; level 4, admitted to hospital but requiring supplemental oxygen; level 5, admitted to hospital requiring non-invasive mechanical ventilation; level 6, admitted to hospital requiring extracorporeal membrane oxygenation, invasive mechanical ventilation (IMV), or both; and level 7, death.

Symptoms or signs collected during the interviews are alopecia, arthromyalgia, asthenia, weight loss, headache, impaired mobility, diarrhoea, peripheral neuropathies, dyspnoea, sexual dysfunctions, behavioural disorders, smell and taste alterations, sleep disorders, mood disorders, fever, cough.

In order to define a clinical homogeneity, we have grouped these symptoms into five macro-categories, hereinafter referred to as "symptoms categories"(SC): respiratory SC (dyspnoea, cough), neurological SC (peripheral neuropathies, headache, impaired mobility, behavioural disorders, cognitive disorders), psychological SC (sleep disorders, mood disorders), muscular disorders (arthromyalgia, asthenia), “other” SC (fever, alopecia, diarrhoea, weight loss, smell and taste alterations, sexual dysfunctions).

- pulmonary evaluation with simple spirometry, 6 min walking test, and calculation of Modified British Medical Research Council (MMRC) dyspnoea scale.
- geriatric evaluation if the patient was previously admitted in AMW, or intensive care evaluation if he was hospitalised in ICU.
- During geriatric and intensive care visits, several questionnaires are administered. In detail, health quality status is evaluated with the EQ5D-5L questionnaire; motor performance with the Short Physical Performance Battery. Cognitive performance is tested with the Montreal Cognitive Assessment and nutritional status with the Mini Nutritional Assessment (MNA). Regarding psychological status, we use PTSS-10 and HADS for patients younger than 65 years while GAD and Geriatric Depression Scale for those older than 65 years. Moreover, we evaluate patients’ frailty with the nine-point-based CFS: a score of 1 indicates a very fit person, while a score of 9 indicates a terminally ill person.
- nursing evaluation to check cutaneous lesions, pressure ulcers, and scarring outcomes.

On the second day, all specialists involved in this project meet for a joint evaluation to identify the clinical problems and the therapeutic pathways for each patient. Teamwork is coordinated by the infectious disease specialist.

On the third day, a week after the first visit, the infectious disease specialist reports the multidisciplinary evaluation to the patient and provides recommendations for follow-up.

All the data are collected through an electronic Case Report Form, implemented in the RedCapCloud platform.

### Statistical analysis

Patients’ characteristics were described using median and interquartile range (IQR), if in a continuous scale, or frequency and percentage, otherwise.

Association between patients’ characteristics and presence of SC at follow-up visit was estimated by a logistic multivariable regression model. The included regressors were gender, age, BMI and presence of comorbidities at diagnosis, ICU admission and presence of disorders at discharge. The model was adjusted for the time between discharge and follow-up visit, to take into account different follow-up times. Patients’ characteristics effects were reported in terms of odds ratios (OR) and 95% confidence intervals (95% CI). P-values were considered statistically significant if lower than 0.05.

All analyses were performed using SAS software version 9.4 (SAS Institute, USA).

## Results

From June 2020 to July 2021, 1059 patients discharged after COVID-19 hospitalization were contacted to invite them to a follow-up visit. Those who accepted (n = 361, 34.1%) were scheduled. A caregiver was allowed to accompany patients with cognitive or physical impairment.

Among the 361 visited patients, 128 were in the ICU group (35.5%) and 233 were in AMW group. 170 AMW patients (73%) had required Continuous Positive Airway Pressure (CPAP), a type of non-invasive ventilation. The median length of hospital stay was 20 days (IQR 13–32); in detail, 38 (IQR 24–49) days for persons in the ICU group and 16 (IQR 11–23) for those in the AMW group. The median time from discharge to the follow-up visit was 128 days (IQR 100–195).

Demographical characteristics are shown in Table 1. The median age was 59 (IQR 53–68) years. One third of patients were over 65 years old. Two hundred and fifty-six (70.9%) patients were male. The median BMI at hospital admission was 27.8 (IQR 25.4–31.2). Almost all (353 of 361) patients had available information on BMI, one hundred and fifty-five (43.9%) were overweight and 105 (29.7%) had first-grade obesity.

**Table 1:** Characteristics of 361 patients at hospital admission, discharge, and follow-up visit

	Patients’ characteristics	Total (n = 361)
Hospital admission	Age (years), median (IQR)	59 (53–68)
	< 65 years	231 (64.0)
	≥ 65 years	130 (36.0)
	Sex	
	Male	256 (70.9)
	Female	105 (29.1)
	BMI (Kg/m^2^) (n = 353), median (IQR)	27.8 (25.4–31.2)
	< 18.5	1 (0.3)
	18.50–24.99	78 (22.1)
	25–29.99	155 (43.9)
	30–39.99	105 (29.7)
	40 +	14 (4.0)
	Smoking history (n = 338)	
	Ever smoker	176 (52.1)
	Never smoker	162 (47.9)
	Education (years) (n = 304), median (IQR)	11 (8–13)
	Comorbidities (n = 336)	
	1 comorbidity	112 (33.3)
	2 comorbidities	135 (40.2)
	> 2 comorbidities	66 (19.6)
	Hypertension	143 (42.6)
	Myocardial infarction	19 (5.7)
	Peripheral vascular disease	18 (5.4)
	Solid tumors	14 (4.2)
	Diabetes	41 (12.2)
	Clinical Score frailty (n = 318)	
	1	105 (33)
	2	150 (47.2)
	3	51 (16.1)
	4	7 (2.2)
	5	3 (0.9)
	6	2 (0.6)
Discharge	Admitted to ICU	128 (35.5)
	Severity scale* (n = 305)	
	3	4 (1.3)
	4	38 (12.5)
	5	170 (55.7)
	6	93 (30.5)
	Days of hospital stay (n = 325), median (IQR)	20 (13–32)
	Therapies at discharge	
	Steroids (n = 357)	107 (30.0)
	Anticoagulant therapy (n = 361)	69 (19.1)
	Oxygen therapy (n = 361)	68 (18.8)
Follow-up visit	Days from discharge to follow-up visit, median (IQR)	128 (100–195)
	Clinical Score frailty (n = 312)	
	1	55 (17.6)
	2	135 (43.3)
	3	89 (28.5)
	4	22 (7.1)
	5	4 (1.3)
	6	6 (1.9)
	7	1 (0.3)

Data are reported as N (%) if not otherwise specified

*BMI* Body Mass Index, *ICU* Intensive care Unit

*Severity scale: 1, not admitted to hospital with resumption of normal activities; 2, not admitted to hospital, but unable to resume normal activities; 3, admitted to hospital but not requiring supplemental oxygen; 4, admitted to hospital but requiring supplemental oxygen; 5, admitted to hospital requiring high-flow nasal cannula (HFNC), non-invasive mechanical ventilation (NIV), or both; 6, admitted to hospital requiring extracorporeal membrane oxygenation, invasive mechanical ventilation (IMV), or both; and 7, death

Three hundred and thirty-six patients had information about comorbidities (see comorbidities’ details in Table 1). The most common comorbidities were hypertension (143/336, 42.6%), followed by diabetes (41/336, 12.2%). Two hundred and one persons (59.8% of 336) had two or more comorbidities (see Table 1).

The CSF obtained at hospital admission was available for 318 patients. Of these 306 (96%) had a CSF score lower than 4, suggesting the absence of vulnerability or frailty.

Thirty percent of patients were discharged from the hospital with the prescription of steroids, 19% with anticoagulant therapy, and 19% with oxygen.

**Fig. 2:**
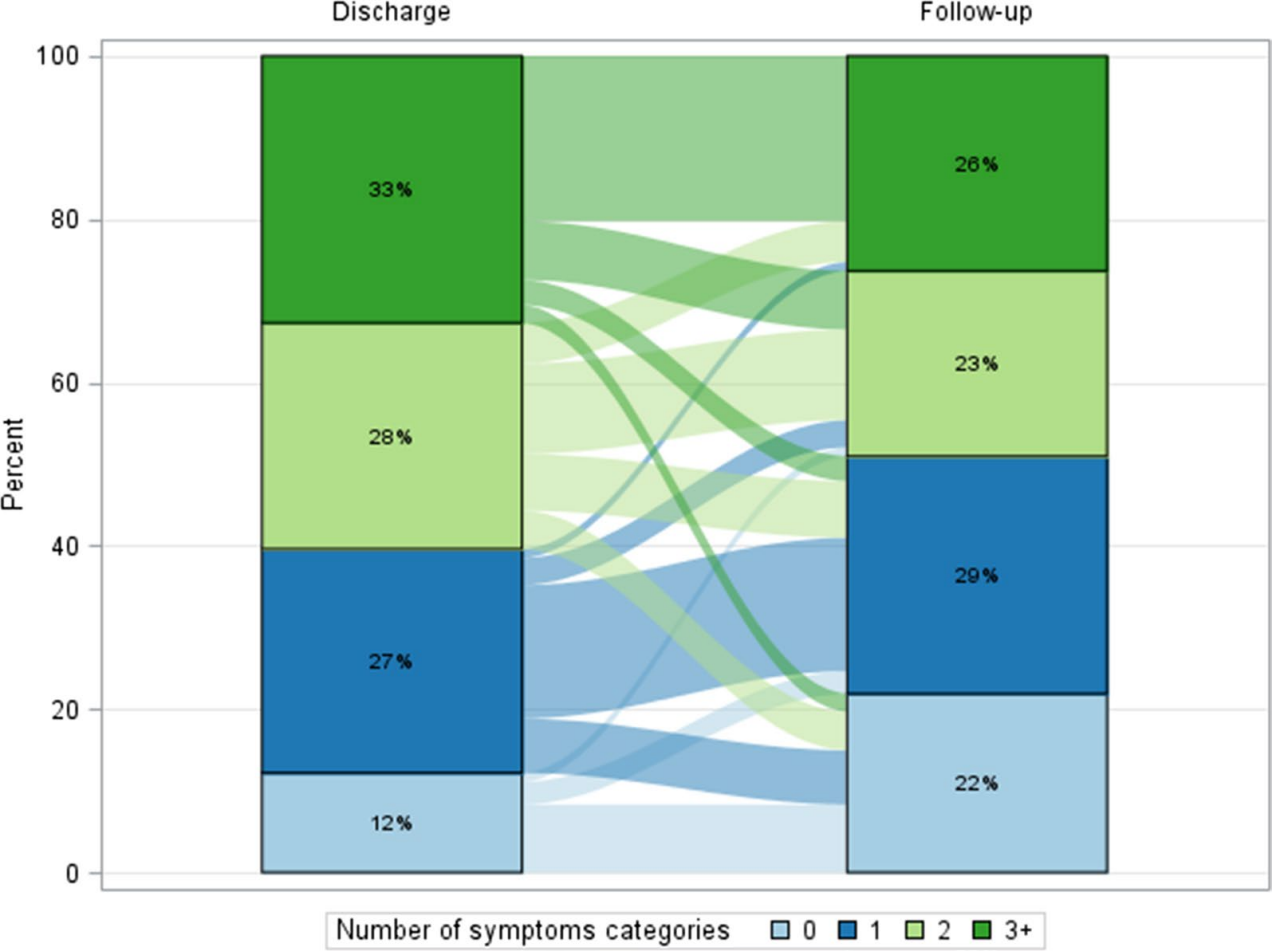
Alluvional plot with percentage of symptoms categories at baseline and at follow up

Patients with at least one SC at discharge were 317/361 (87.8%) while 243/361 (67.3%) had persistence of SC at follow-up (see Table 2). Moreover, in 159/361 patients (44%) new SC were involved during follow up: 116 (72.9%) one SC, 39 (24.5%) two SC, 4 (2.5%) three or more SC. At follow up visit 130 of 159 (82%) were still with symptoms (see Table 2). A statistically significant difference were found between the number of SC at baseline and at follow-up (p < 0.001). A high fluctuation of involved SC was observed between discharge and follow-up (see Fig. 2). In detail, 32.7% of the sample reported 3 SC at discharge; of these patients, only 61.2% continued to report 3 SC while the rest of the sample reduced the number of SC at follow-up visit.

**Table 2:** Number of Symptoms Categories (SC) involved at discharge vs number of SC at follow up visit compared to number of SC occurring during follow-up

Number of SC	N. of patients with SC at discharge	Number of patients by SC present at discharge still present at follow-up			
		0	1	2	≥ 3
0	44 (12.2)	44 (100.0)	0 (0.0)	0 (0.0)	0 (0.0)
1	99 (27.4)	41 (41.4)	58 (58.6)	0 (0.0)	0 (0.0)
2	100 (27.7)	24 (24.0)	33 (33.0)	43(43.0)	0 (0.0)
≥ 3	118 (32.7)	9 (7.6)	12 (10.2)	37 (31.4)	60 (50.8)
TOTAL	361 (100)	118 (32.7)	103 (28.5)	80 (22.2)	60 (16.6)

	N. of patients with SC developed during follow up	Number of patients by SC developed during follow-up, still present at follow-up visit			
		0	1	2	≥ 3
0	202	202 (100.0)	0 (0.0)	0 (0.0)	0 (0.0)
1	116	26 (22.4)	90 (77.6)	0 (0.0)	0 (0.0)
2	39	3 (7.7)	3 (7.7)	33 (84.6)	0 (0.0)
≥ 3	4	0 (0.0)	0 (0.0)	2 (50.0)	2 (50.0)
TOTAL	361	231 (64.0)	93 (25.8)	35 (9.7)	2 (0.5)

Difference in SC at discharge and follow up was statistically significant (Chi-square test p-value < 0.0001)

Table 3 describes the factors associated with the presence of PCC disorders. In a regression model, male gender (OR 3.23, CI 95% 1.46–7.15; p = 0.004), ICU admission (OR 2.78, CI 95% 1.29–5.96, p = 0.009), and presence of SC at discharge (OR 14.39, CI 95% 6.41–32.32, p < 0.001) were associated with the risk of having disorders at follow-up. BMI, evaluated both as a continuous variable (OR 1.12, CI 95% 1.04–1.21, p = 0.005 Model A) and as a categorical one (Model B), was also a risk factor for PCC. No associations with time from discharge, comorbidities, and age were observed.

**Table 3:** Multivariable logistic regression models on presence of Symptoms Categoires (SC) at follow-up visit

Model A: N = 331	Odds ratio (95% CI)	P-value
Male (vs Female)	3.23 (1.46–7.15)	0.004
Age (by 1 year)	1.01 (0.99–1.04)	0.340
BMI (by 1 unit)	1.12 (1.04–1.21)	0.005
ICU admission (vs No)	2.78 (1.29–5.96)	0.009
Presence of comorbidities (vs No)	1.35 (0.65–2.80)	0.420
Time between discharge and follow-up visit (by 1 day)	1.00 (0.99–1.00)	0.676
Presence of disorders at discharge vs No	14.39 (6.41–32.32)	< 0.001

Model B: N = 331	Odds ratio (95% CI)	P-value
Male (vs Female)	3.29 (1.48–7.29)	0.003
Age (by 1 year)	1.01 (0.98–1.04)	0.404
BMI overweight (vs normal weighted)	1.85 (0.89–3.84)	0.098
BMI obese (vs normal weighted)	3.32 (1.41–7.83)	0.006
ICU admission (vs No)	2.81 (1.31–6.04)	0.008
Presence of comorbidities (vs No)	1.39 (0.67–2.88)	0.374
Time between discharge and follow-up visit (by 1 day)	0.99 (0.99–1.00)	0.687
Presence of disorders at discharge vs No	14.13 (6.33–31.53)	< 0.001

BMI overweight defined as ≥ 25 and < 30; BMI obese defined as ≥ 30. Model A: Body Mass Index (BMI) as continuous variable; Model B: BMI as categorical variable

*BMI* Body Mass Index, *ICU* Intensive care Unit

## Discussion

This paper aims to describe our model of a multidisciplinary approach to PCC. This model of care has an important advantage: it completely and readily identifies the patients’ clinical needs, simultaneously considering their engagement and the organization of care pathways. For this reason, the idea behind our approach was to reverse the usual practice where the patients must adapt to a rigidly predefined organization. In our model of care, the patients with PCC enter a guided multidisciplinary care pathway, modelled on their clinical needs, and conceived to manage both clinical and organizational aspects in the best possible manner.

Patients are visited vis-a-vis and can perform, on the same day, three specialistic visits at least (with the infectious disease specialist, the pneumologist, and the geriatrician or intensive care medical doctor) and all the tests necessary to evaluate disorders potentially associated with PCC. They also receive written information and advice about PCC and complete a set of psychological questionnaires to evaluate anxiety, depression, and Post Traumatic Stress Disorder. Caregivers, if present, are also involved in this experience, filling in dedicated questionnaires and participating in symptoms description, to integrate the information reported by the patients.

Our methodology, although developed at the beginning of the COVID-19 pandemic, has many points in common with one of the most recent definitions of symptoms after COVID-19 [12]. WHO defined PCC as conditions that occur “in individuals with a history of probable or confirmed SARS-CoV-2 infection, usually 3 months from the onset of COVID-19 with symptoms that last for at least 2 months and cannot be explained by an alternative diagnosis [12]. We focused our attention on signs and symptoms at a median time of 128 days from discharge with a lower quartile of 100 days.

Moreover, in accordance with NICE guidelines recommendations, in our PCC clinic, the older people also underwent a multidimensional geriatric assessment.

Our model of care is also comparable to the one proposed by Parker et al. [10] with a holistic approach and evaluation for an indication for further instrumental exams and visits. Moreover, using this approach, patients are prospectively assessed allowing a better definition of the clinical disorders associated with PCC. Much research in this field was conducted through cross-sectional surveys and was therefore at high risk of bias, due to the retrospective nature of the studies. Often the data were self-reported and consequently prone to recall bias.

The clinical findings from data collected using this approach are multiple and under development. In this preliminary article, we only focused on the methodology, the baseline characteristics of evaluated patients, and the frequency of complained disorders.

We observed that the number and type of symptoms fluctuated from discharge to follow-up, confirming that recovery could be illusory, or that new SC could be involved [20]. For example, at discharge, 12.2% of people did not report any SC, but during follow-up 159 patients (44.03%) developed at least one SC. Moreover, 130 patients (36%) still complained SC developed during follow-up. These data further support the need for a multidisciplinary approach to PCC.

The percentages of acquisition of and recovery from SC are extremely variable (see Fig. 2). On the other hand, the prevalence of symptoms reported in the literature was extremely variable according to the time of evaluation from the discharge and the proportion of people admitted to ICU [21, 22]. We found that ICU admission could be one of the causes for the presence of SC at follow-up and this could be due to the partial overlap between Post Intensive Care Syndrome (PICS) and PCC.

We did not find a relationship with time between discharge and follow-up visit, partially confirming the fluctuation of disorders as shown by our results.

Comorbidities did not seem associated with the presence of symptoms at follow-up, suggesting a new-onset syndrome not related to previous pathological conditions.

In our analysis, overweight and obesity were associated with the presence of symptoms at follow-up, confirming data from the literature [23, 24].

We found an association with male sex that was in contrast with previous studies [4, 25], thus further analyses on larger samples are needed to validate this finding. However, we can argue that female sex is usually affected by depressive symptoms independently of COVID and this could be a selection bias that was absent in our sample, because of the small proportion of women. Moreover, unlike the cohort described by Huang [4], in our population, a high percentage of patients underwent CPAP or mechanical ventilation. As it has been shown by several papers [26, 27], the male sex is associated with the risk of developing more severe forms of COVID. In our cohort, most of the patients were male and with a severe degree of disease and this may have affected this association.

Our study has some limitations. A high proportion of patients was in the ICU group. This may have partially biased our results since we cannot rule out that some patients may have had PICS and not only (or exclusively) PCC. Second, as we do not have a control group, matched for age and comorbidities, we cannot estimate the net burden of PCC. Finally, since initially we enrolled patients with a more severe disease, PCC may be partially overestimated. For this reason our results could not be extended to all patients that were affected by SARS-CoV-2 infection but only to patients that were hospitalized for COVID-19.

In conclusion, our model of care is in accordance with the most recent guidelines for the management of PCC. Our results show that symptoms of PCC are highly variable and fluctuating in a population of patients with severe COVID-19..

Obesity, ICU admission, and symptoms at baseline are risk factors for the presence of symptoms at the follow-up visit.

## Data Availability

The datasets generated during and/or analyzed during the current study are not publicly available to protect our participants’ sensitive data but are available from the corresponding author on reasonable request.

## Abbreviations

SARS: Severe Acute Respiratory Syndrome
CoV: CoronaVirus
COVID-19: Corona Virus Disease 2019
PCS: Post-COVID-19 syndrome
PASC: Post-acute sequelae of SARS-CoV-2 infection
NICE: National Institute for Health and Care Excellence
WHO: World Health Organization
PCC: Post-COVID-19 conditions
EQ5D-5L: Euro Quality- 5 Dimension-5 Level
CFS: Clinical frailty score
ICU: Intensive care unit
AMW: Acute medical wards
BMI: Body mass index
PTSS: Post-traumatic symptom scale
HADS: Hospital anxiety and depression scale
GAD: Generalized anxiety disorder assessment
IMV: Invasive mechanical ventilation
MMRC: Modified British Medical Research Council
IQR: Interquartile range
OR: Odds ratios
CPAP: Continuous positive airway pressure
PICS: Post intensive care syndrome
